# Case report of a duplicated cystic duct: A unique challenge for the laparoscopic surgeon

**DOI:** 10.1016/j.ijscr.2019.02.030

**Published:** 2019-02-28

**Authors:** Semeret Munie, Hassan Nasser, Pauline H. Go, Kelly Rosso, Ann Woodward

**Affiliations:** a Department of Surgery/Division of Trauma and Acute Care Surgery, Henry Ford Hospital, 2799 W Grand Blvd, Detroit, MI, 48202, USA; b Department of Surgery, Henry Ford Hospital, 2799 W Grand Blvd, Detroit, MI, 48202, USA; c Department of Cardiothoracic Surgery, University of Iowa, 200 Hawkins Drive, Iowa City, IA, 52242, USA; d Department of Surgical Oncology, Banner MD Anderson, 14416 W Meeker Blvd, Sun City West, AZ, 85375, USA

**Keywords:** ERCP, endoscopic retrograde cholangiopancreatography, IOC, intraoperative cholangiogram, Duplicated cystic duct, Double cystic duct, Laparoscopic cholecystectomy, Bile duct variation

## Abstract

**Introduction:**

Anatomical variants of the extrahepatic biliary tree are numerous, adding significantly to the risk of bile duct injury during cholecystectomy, especially when laparoscopic approach is employed. Duplicated cystic ducts draining a single gallbladder are extremely rare.

**Presentation of case:**

A 34-year-old female presented with signs and symptoms of acute cholecystitis which was confirmed on imaging. She was found to have an accessory cystic duct on laparoscopic cholecystectomy requiring conversion to open laparotomy with intraoperative cholangiogram to delineate the anatomy.

**Discussion:**

In the English literature, there has been 20 reported cases of double cystic duct with a single gallbladder. Most of these cases were diagnosed intraoperatively despite the completion of a preoperative endoscopic retrograde cholangiopancreatography in a few of these patients.

**Conclusion:**

The limited success of preoperative biliary tract imaging in demonstrating anatomic aberrancies prior to cholecystectomy clearly highlights the importance of maintaining constant vigilance for even the slightest anatomic abnormality at operation. Any uncertainty or concern for ductal injury mandates immediate operative cholangiogram with cannulation of all structures in question.

## Highlights

• Double cystic ducts with a single gallbladder is exceedingly rare.
• Diagnosis of this anatomic variant is most commonly made intraoperatively.
• Knowledge of biliary aberancies is crucial to preventing bile duct injury.
• IOC should be utilized to elucidate biliary anatomy when unclear.

## 1 Introduction

Biliary tree anomalies have been detected in up to 47% of the population based on operative, cholangiographic and autopsy studies [1]. The basis of bile duct injury is failure to identify biliary anatomy especially in the cases aberrancies. Thus, Identification of these anomalies in biliary anatomy is crucial to avoid the morbidity and mortality associated with bile duct injuries. Unlike the more common variant in which two cystic ducts drain two distinct gallbladders or cavities [2], duplicated cystic ducts draining a single, unilocular gallbladder is extraordinarily rare, with fewer than 20 cases reported in the English literature. We report our experience with laparoscopic cholecystectomy in the setting of double cystic ducts identified intraoperatively. The case report has been reported in line with the surgical case report (SCARE) criteria [3].

## 2 Presentation of case

We report a case of a 34-year-old female who presented to the emergency department with three days of constant right upper quadrant and epigastric abdominal pain with associated nausea. The patient had similar pain two months ago that resolved and did not seek medical attention. On presentation she had normal vital signs. Physical examination demonstrated right upper quadrant tenderness without peritoneal signs with negative clinical Murphy’s sign. Her blood work revealed a white blood cell count of 13,500 /microliter, alanine aminotransferase of 318 U/L, aspartate aminotransferase of 259 U/L, alkaline phosphatase of 120 U/L, and total bilirubin of 1.7 mg/dL. Ultrasonography demonstrated new mild gallbladder wall thickening and negative sonographic Murphy’s sign with equivocal suggestions of acute cholecystitis. Hepatobiliary iminodiacetic acid scan was subsequently performed and showed non-visualization of the gallbladder consistent with acute cholecystitis.

**Fig. 1:**
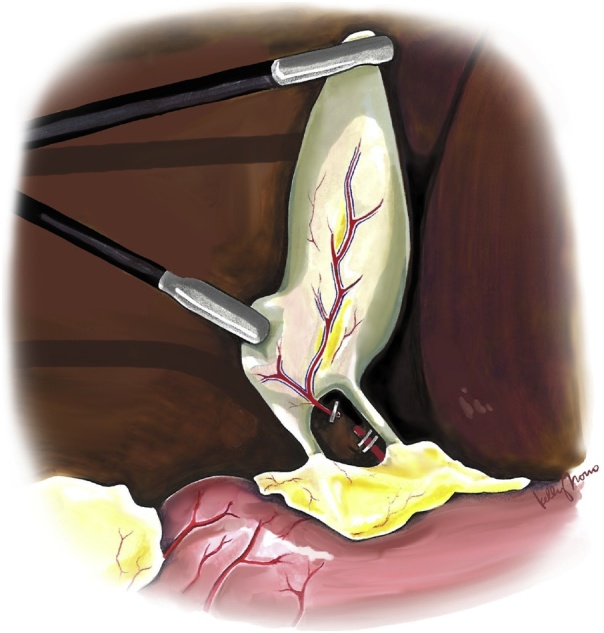
Schematic demonstration of the visualized anatomy intraoperatively.

**Fig. 2:**
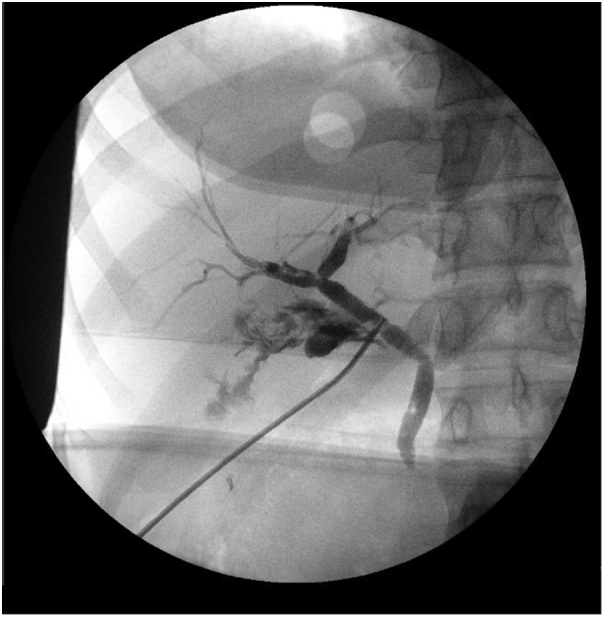
Intraoperative cholangiogram through main cystic duct showing correct ductal anatomy with intact common bile, common hepatic, as well as right and left hepatic ducts.

**Fig. 3:**
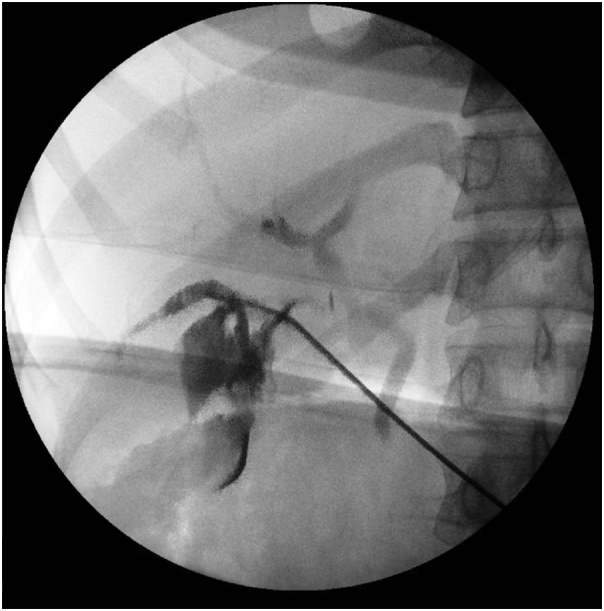
Cholangiogram through accessory duct failing to fill bile duct due to blocked proximal aspect of lumen.

The patient was taken to the operating room and the cystic duct and artery were dissected free from the cystic triangle laparoscopically. Both structures were secured proximally and distally and divided sharply. The gallbladder was dissected from the bed using electrocautery. Due to the contracted and intrahepatic nature of the gallbladder, approach was switched to a top-down technique. Just prior to removal of the gallbladder from the liver bed, another structure entering the gallbladder was encountered (Fig. 1). At this point due to abnormal anatomy and inadequate visualization, the decision was made to convert to an open procedure. On further evaluation, the structure appeared to be a bile duct. Intraoperative cholangiogram was performed through the cystic duct that had been clipped earlier, which showed correct ductal anatomy with intact CBD, common hepatic, as well as right and left hepatic ducts (Fig. 2). An attempt was made to cannulate the second duct for cholangiogram and bile return was noted from the duct. However, during cholangiography contrast extravasated outside rather than filling the bile duct, due to impacted stones blocking proximal aspect of the duct (Fig. 3). The second accessory duct was clipped and transected and the gallbladder was removed. The gallbladder was evaluated and showed the two cystic ducts with distal open lumens that communicated to the gallbladder.

Postoperatively, the patient’s liver function tests normalized. Patient was discharged home on postoperative day 3 and was tolerating diet. She was seen in the surgical clinic 2 weeks postoperatively and was doing well. The pathology report showed acute on chronic cholecystitis with mucosal ulceration and cholelithiasis.

## 3 Discussion

Intrigue with anomalous gallbladder anatomy and associated extrahepatic biliary duct aberrancy originates as far back as 1926 with Edward Boyden’s comparative report and classification of the various congenital anomalies of the gallbladder [4]. Thirty years later, Caster and Flannery categorized cystic duct duplication into 3 types: (1) "Y" type, wherein 2 cystic ducts join to form a single cystic duct that then enters the CBD, (2) "H" type, in which each cystic duct independently joins the bile duct system at the CBD, right hepatic duct, left hepatic duct or common hepatic duct, and (3) trabecular type, in which one cystic duct enters the CBD while the other directly enters the liver parenchyma [5].

In the English literature, there has been 20 reported cases of duplicate cystic ducts draining a single gallbladder including our reported case (Table 1) [1,2,[6], [7], [8], [9], [10], [11], [12], [13], [14], [15], [16], [17], [18], [19], [20], [21]]. Females constituted 75% of the reported cases. “H” type duplication with the cystic ducts joining the biliary system at the CBD, common hepatic duct, or right hepatic duct was reported in 11 cases (55%), representing the most common configuration. The “Y” and trabecular types represent 30% and 15% of the reported cases to date, respectively.

**Table 1:** Cases of duplicate cyst ducts draining a single gallbladder reported in the English language literature.

Case	Author	Age/gender	Country	Duplication type	Preoperative ERCP	Diagnosis	Operative approach	IOC
1	Perelman 1961 [4]	56/Female	USA	“H” type	No	Intraoperative	Not reported	Not reported
2	Senapati and Wolf 1984 [5]	56/Male	UK	Trabecular type	No	Intraoperative	Open	Yes
3		55/Female		“H” type	No	Intraoperative	Open	Yes
4	Nakasugi et al 1995 [8]	50/Female	Japan	“Y” type	Yes	Preoperative ERCP	Laparoscopic	Yes
5	Ng et al 1996 [3]	60/Male	Hong Kong	“H” type	Yes	Intraoperative	Laparoscopic converted to open	No
6	Momiyama et al 1996 [9]	66/Female	Japan	“H” type	Yes	Postoperative	Laparoscopic	Yes
7	Hirono et al 1997 [10]	74/Female	Japan	“H” type	Yes	Intraoperative	Laparoscopic	Yes
8	Fujikawa et al 1998 [1]	70/Female	Japan	“H” type	Yes	Intraoperative	Open	Yes
9	Lobo et al 2000 [11]	49/Female	Brazil	“Y” type	No	Intraoperative	Laparoscopic	Yes
10	Tsutsumi et al 2000 [12]	74/Female	Japan	“H” type	Yes	Preoperative ERCP	Laparoscopic	Yes
11	Shivhare et al 2002 [13]	46/Female	India	“H” type	No	Intraoperative	Laparoscopic converted to open	Yes
12	Huston et al 2008 [14]	43/Female	USA	“H” type	No	Intraoperative	Laparoscopic	Yes
13	Aristotle et al 2011 [15]	50/Male	India	“Y” type	NA	Postmortem	NA	NA
14	Shih et al 2011 [16]	37/Male	Taiwan	“Y” type	No	Intraoperative	Laparoscopic	No
15	Shabanali et al 2014 [17]	50/Female	Iran	“H” type	No	Intraoperative	Laparoscopic	No
16	Otaibi et al 2015 [18]	54/Male	USA	“H” type	No	Intraoperative	Laparoscopic	Yes
17	Samnani et al 2015 [19]	34/Female	Pakistan	“Y” type	No	Intraoperative	Laparoscopic	No
18	Fujii et al 2017 [20]	57/Female	Japan	Trabecular type	Yes	Preoperative ERCP	Laparoscopic	Yes
19	Salih et al 2017 [21]	33/Female	Iraq	“Y” type	No	Intraoperative	Laparoscopic	No
20	Present case	34/Female	USA	Trabecular type	No	Intraoperative	Laparoscopic converted to open	Yes

Abbreviations: ERCP, endoscopic retrograde cholangiopancreatography; IOC, intraoperative cholangiogram.

Double cystic duct was identified intraoperatively in 16 out of the 19 patients (84%) who were operated on. Despite the completion of a preoperative endoscopic retrograde cholangiopancreatography (ERCP) in 7 patients, the cystic duct anomaly was only identified in 3 cases (43%) [1,2,[8], [9], [10],^12^,^20^]. This emphasizes the importance of being aware of this anatomic variant as even with invasive preoperative testing, the accessory duct was only identified intraoperatively. Cholecystectomy was performed and completed laparoscopically in 12 cases and intraoperative cholangiogram (IOC) was performed in 8 of these cases to delineate the anatomy when a second cystic duct was encountered [[8], [9], [10], [11], [12],^14^,[16], [17], [18], [19], [20], [21]]. Three other cases required conversion to laparotomy, one of which was our case, and was due to inability to clearly define biliary anatomy laparoscopically [2,13]. One case required reoperative laparotomy due to delayed recognition of the duplicated cystic duct, resulting in bile leak [9]. An IOC and preoperative ERCP was performed in that case but did not prevent the complication of a biliary leak.

## 4 Conclusion

The limited success of preoperative biliary tract imaging in demonstrating anatomic aberrancies prior to cholecystectomy clearly highlights the importance of maintaining constant vigilance for even the slightest anatomic abnormality at operation. Any uncertainty or concern for ductal injury mandates immediate operative cholangiogram with cannulation of all structures in question. Although laparoscopic cholecystectomy is safe in experienced hands, a low threshold for conversion to laparotomy should be had when the anatomy cannot be deciphered.

## Conflicts of interest

No conflicts of interest to be declared.

## Funding

No source to be stated.

## Ethical approval

The study is exempt from ethical approval in our institution.

## Consent

Written informed consent was obtained for publication of this case report and accompanying images. A copy of the written consent is available for review by the Editor-in-Chief of this journal on request.

## Author contribution

Semeret Munie: Formal analysis; Writing – original draft.

Hassan Nasser: Writing – review & editing.

Pauline H. Go, MD: Writing – original draft.

Kelly Rosso, MD: Visualization.

Ann Woodward: Supervision.

## Registration of research studies

Not applicable.

## Guarantor

Ann Woodward, MD.

## Provenance and peer review

Not commissioned, externally peer reviewed.
